# Nutritional Composition of Beach-Cast Marine Algae from the Brazilian Coast: Added Value for Algal Biomass Considered as Waste

**DOI:** 10.3390/foods11091201

**Published:** 2022-04-21

**Authors:** Andrea Mandalka, Maria Irisvalda Leal Gondim Cavalcanti, Talissa Barroco Harb, Mutue Toyota Fujii, Peter Eisner, Ute Schweiggert-Weisz, Fungyi Chow

**Affiliations:** 1 ZIEL-Institute for Food & Health, TUM School of Life Sciences Weihenstephan, Technical University of Munich, 85354 Freising, Germany; peter.eisner@ivv.fraunhofer.de; 2 Fraunhofer Institute for Process Engineering and Packaging IVV, Giggenhauser Str. 35, 85354 Freising, Germany; ute.weisz@ivv.fraunhofer.de; 3 Instituto de Botânica, Av. Miguel Estéfano 3687, São Paulo 04301-902, Brazil; iriscavalcanti@ifpi.edu.br (M.I.L.G.C.); mutue.fujii@gmail.com (M.T.F.); 4 Institute of Bioscience, University of São Paulo, Rua do Matão 321, São Paulo 05508-090, Brazil; talissaharb@hotmail.com; 5 Faculty of Technology and Engineering, Steinbeis-Hochschule, George-Bähr-Str. 8, 01069 Dresden, Germany; 6 Institute for Nutritional and Food Sciences, University of Bonn, 53115 Bonn, Germany

**Keywords:** amino acids, fatty acids, food, functional ingredient, raw material, seaweeds

## Abstract

In some coastal areas, large quantities of beach-cast macroalgae can accumulate and are usually considered waste and disposed of. However, due to their biofunctional and nutritional properties, they have great potential as a new source of raw materials. Increasing population growth has made the search for alternative raw materials with valuable nutritional properties urgent; here, beach-cast macroalgae could provide great potential. Our research goal was to characterize the nutritional profile of 12 beach-cast seaweed species from the Brazilian coast to assess their potential valorization. A considerable number of nutritional compounds was observed, such as ash (6.5–59.3%), total dietary fibers (22.1–65.8%), proteins (5.1–21.5%), and carbohydrates (31.4–81.0%), with an expressive abundance of minerals, free amino acids, and fatty acids. *Spatoglossum schroederi* and *Alsidium seaforthii* showed protein contents of 21.5 ± 0.2%, 19.7 ± 0.1%, and high amounts of total dietary fiber of 59.2 ± 0.4%, 61.7 ± 4.9%, respectively. The overall profile suggests that beach-cast seaweeds are suitable for nutritional and other bioeconomical purposes, to which different species with different characteristics contribute. Contamination of these seaweeds with unwanted toxic compounds like micropollutants was not studied. However, this must be considered before they are used for human consumption.

## 1. Introduction

Population growth together with increasingly limited or overused arable lands and freshwater resources has led to the need for alternative protein sources and raw materials with valuable nutritional properties. Presently, plant proteins are primarily produced by land crops. Macroalgae, which do not compete with traditional food crops for agricultural land, is still underutilized and could provide a valuable, fast-growing protein source together with other nutritious components [1,2,3,4].

Several studies have shown that macroalgae have an interesting nutritional and chemical composition; particularly, red and green seaweed species are gaining interest as protein-rich foods for human consumption and as sources of protein biofunctional peptide components [5,6,7,8]. Seaweeds may contain up to 47% of protein on a dry weight (dw) basis, comparable to those of soybean (47–52%) and lupine (39–55%) [9,10]. They are increasingly recognized as a natural source of proteins, dietary fibers, polysaccharides, polyunsaturated fatty acids (PUFA), minerals, vitamins, pigments, and phytochemicals, such as polyphenols [11,12,13].

In addition, it is known that the chemical composition of macroalgae as well as their nutritional and medicinal value depends on many factors, such as species and their development stages, geographic origin or growing area, habitat, season, environmental conditions, time of harvest, and processing methods, such as various sampling procedures and drying methods [14,15,16,17,18]. Many studies have revealed that their potentially bioactive peptides might have protective effects against allergies, cancer, cardiovascular disease, degenerative diseases, diabetes, digestive disorders, hypertension, inflammation, obesity, and oxidative stress [17,19].

In 2019, seaweed cultivation production increased to 35.8 million tons, which account for 97% of the world’s seaweed production (FAO 2021) [20]. The global use of macroalgae-derived products is now a multi-billion dollar industry. These products are mainly techno-functional polysaccharides, for example, the phycocolloids agar, carrageenans, and alginates for food, cosmetic, and pharmaceutical industries, or fertilizers and feed ingredients. As there are only a few macroalgae species suitable for aquaculture production, and predatory extractive harvesting from natural stocks is no longer a globally accepted practice, many researchers and companies have now focused on the search for new species for aquaculture, natural seabed management, or other available seaweed materials [21,22].

Many studies [23,24,25,26] report the potential nutritional or bioactive content of various algae species worldwide, but there are only a few studies on Brazilian beach-cast algae. Their chemical composition has not been sufficiently investigated [27,28,29,30,31]. Brazil has an insignificant share of the global seaweed market, although the Brazilian coastal area has an excellent potential for collecting beach-harvested seaweeds [27,30,31]. However, every year, tons of beach-cast seaweeds are removed from the beach by local authorities as part of beach cleaning operations and disposed of into landfill sites as part of urban waste [32,33,34]. Despite the richness of the algal flora in Brazil, they are rarely used—only in coastal regions as fertilizer.Since beach-cast algae tend to cover large areas of the coast, harming the local tourism industry and fisheries, their collection could have great potential for exploiting the unused biomass for new marine-related industries in Brazil. Their collection could increase the value of macroalgae, for which there is currently no adequate market price in Brazil. Species richness as well as their abundance are essential information for bioprospecting, uses for beach-cast macroalgae, and the gaps in the studies are pointed out by Harb et al. and Cavalcanti et al. [35,36].

Current production or use of chemicals from seaweeds is focused on a few macroalgae species and individual products, such as hydrocolloids or xanthophylls with little to no use for the remaining biomass. Still, there are still thousands of unexamined species that contain important nutrients such as proteins, minerals, fibres, fatty acids, and other useful bioactives, and could serve as a future alternative food source. Screening the biochemical composition of beach algae would be the first step to determine the potential of these algae for further exploitation [26,37].

This study investigates the nutritional value of Brazilian locally abundant beach-cast seaweeds to evaluate their use as potential biofunctional food ingredients. These findings could also reveal species with a high nutritional value that has not yet been harvested or cultivated [20]. Due to the diversity of algae and the general abundance of species, some species may have a previously unknown potential for value-added ingredients. We selected biomass of twelve abundant beach-cast algal species from the Brazilian coast. We characterized the nutrient profile of the algae to evaluate their potential use as food or other dietary supplements and to assess their potential as a regional and sustainable biomass source. To our knowledge, there are no studies that have previously published proximate composition, dietary fibers, minerals, polyunsaturated fatty acids (PUFA), free amino acids, and protein solubility on the algal species analyzed here. 

Furthermore, beach-cast algae are an underutilized and underestimated valuable biomass that should be considered a sustainable source of bioactive compounds in the future. However, an important aspect for the exploitation of seaweeds as a healthy or functional food ingredient is the need to identify and quantify heavy metals and other toxic compounds such as pesticides, which could be absorbed from the seawater. Contamination of seaweeds with these unwanted toxic compounds depends on habitat or ecology. More studies of heavy metal toxicokinetics are needed and food safety awareness needs to be raised for a beneficial and safe algae consumption [13].

## 2. Materials and Methods

### 2.1. Sample Collection and Species Identification

Twelve different abundant biomass species of red, brown, and green beach-cast macroalgae were collected from the southeast and northeast beaches of the Brazilian coast (Table S1). The seaweeds were collected using systematic sampling, in which only visible healthy individuals were selected. The material was rid of macroepiphytes, washed with abundant tap water, and air-dried under a shade. The pre-dried samples were transported to the laboratory, air circulation oven-dried at 40 °C, and then powdered in a ball mill (MA350, Marconi, Brazil).

Three fresh specimens for each species were separated for exsiccates and deposited in the SPF Herbarium (Phycological Section) at the University of São Paulo and the Herbarium of the Instituto de Botânica, São Paulo (SP), Brazil (Table S1). Taxonomic identity was confirmed by Maria Irisvalda Leal Gondim Cavalcanti and Mutue Fujii, both from the Instituto de Botânica, São Paulo, Brazil.

### 2.2. Proximate Composition

Dry matter content (105 °C), ash content (950 °C), and protein content were determined according to AOAC Official Methods [38,39] by means of a thermogravimetric method (TGA 701 Leco, St. Joseph, MI, USA) and the Dumas combustion method (TruMac N, Leco Instruments, Mönchengladbach, Germany), respectively. The organic nitrogen content was quantified, and the total protein content was calculated using the nitrogen-to-protein conversion factor N × 6.25, since the recent study by Angell et al. [2] showed that species-specific factors are rarely used for algae and most authors resort to the traditional conversion factor of 6.25 to allow comparisons to previous studies [40], despite the fact that many studies show that this factor leads to an overestimation of the protein content in macroalgae [41,42,43]. Therefore, we calculated a more accurate estimate of protein content as 5.13 for brown, 3.99 for red, and 4.24 for green algae, respectively, as shown in supplemental Table S2 [44,45]. Total carbohydrates were calculated by the difference, subtracting ash, moisture, total lipid, and protein contents from 100%. Soluble carbohydrates were obtained using three-time aqueous extraction for 2 h each at 70 °C and determined using the phenol-sulfuric acid method [46] by absorbance read at 490 nm. Soluble carbohydrates were calculated by referring to the galactose standard curve.

*Dietary fiber analysis.* Soluble (SDF), insoluble (IDF), and total (TDF) dietary fibers were determined according to the enzymatic-gravimetric method AOAC 993.19 and 991.43 (AOAC, 2016 as provided by Megazymes International Ireland, Bray County Wicklow, Ireland) Soluble (SDF), insoluble (IDF), and total (TDF) dietary fibers were determined using Fibertec.

*Mineral composition*. Macro (N, P, Ca, K, Mg, and Na) and micro (Fe) elements as well as trace metals (Cd, Cu) were determined by hydrolysis with concentrated HNO_3_ and H_2_O_2_ 30% (*v*/*v*) in a thermal digester block (DigiPrep, SCP Science, Champlain, USA) and an Inductively Coupled Plasma Optical Emission Spectrometry technique (ICP-OES Spectro Arcos, Spectro Analytical Instruments GmbH, Kleve, Germany).

*Free amino acids.* A free amino acid profile was analyzed according to Santa-Catarina, et al. [47] with some modifications. Samples were extracted using 6 mL ethanol (*v*/*v*) 80% for 2 h, and the supernatants were concentrated under a speed-vacuum. The concentrated sample was re-suspended in 2 mL ultrapure water. The suspension was filtrated using a 0.2 µm Millipore membrane. Amino acids were derivatized with o-phthaldialdehyde (OPA) and identified through HPLC (Shimadzu Shin-pack CLC ODS) using a C18 reverse-phase column (Supelcosil LC-18, 25 cm × 4.6 mm/L × i.d.). The gradient was developed by mixing increasing proportions of 65% methanol to a buffer solution (50 mM sodium acetate, 50 mM sodium phosphate, 20 mL/L methanol, 20 mL/L tetrahydrofuran, and pH 8.1 adjusted with acetic acid). The gradient of 65% methanol was programmed according to Egydio et al. [48]. Fluorescence excitation and emission wavelengths were 250 nm and 480 nm, respectively. Peak areas and retention times were measured by comparison with known quantities of standard amino acids (Sigma-Aldrich, Louis, MO, USA).

*Fatty acids.* Fatty acid content was determined according to the Büchi Caviezel method, where the measurements of the fatty acids were based on a gas chromatographic separation (Agilent 7890A GC system, Agilent Technologies, Waldbronn, Germany, column ZB FFAP Phenomenex, length 15 m × 0.25 mm × 0.25 µm), and detection by a flame ionization detector. The method considers the free and bonded fatty acids from C4 to C24 with a content of 0.1 to 100% regarding the total content of fatty acids in the sample expressed as triglycerides. A defined amount of oil was saponified with potassium hydroxide. Potassium salts of the fatty acids were converted to their free fatty acids by the addition of sodium hydrogen phosphate. Free fatty acids were quantified by gas chromatographic analysis (carrier gas hydrogen 5.0; combustible gases hydrogen and, synthetic air, start temperature 160 °C, temperature gradient 25 °C/min., end temperature 250 °C). The quantification of the total fat content is based on the ratio between the sum of the peak areas of the detected fatty acids and the peak area of the internal standard.

### 2.3. Protein Solubility

Protein solubility (%) was determined in duplicate according to a standardized method based on Morr et al. [49] for pH values from two until 13. For each measurement, a 1.5-g macroalgae sample was suspended in 50 mL 0.1 M NaCl, and the pH was adjusted with 0.1 M NaOH or 0.1 M HCl, respectively. After stirring for 1 h at room temperature, the non-dissolved fractions of the samples were separated by centrifugation (20,000× *g*, 15 min, room temperature), and the supernatants were passed through a Whatman No. 1 filter paper to remove any remaining particulates. The protein content of the supernatant was determined following the Dumas method described in Section 2.2.

### 2.4. Statistical Analysis

The analytical determinations were conducted at least in triplicate, except for protein solubility, which was conducted twice. Values were expressed as mean ± standard deviation (SD) in percentage based on a dry weight (dw) content unless stated otherwise. One-way Analysis of Variances (ANOVA) was conducted followed by a Student–Newman–Keuls (SNK) test to determine the significant differences (*p* < 0.05) among the samples using the software Statistic v.10, by StatSoft, Hamburg, Germany. Additionally, pairwise multiple comparisons with a Euclidean cluster based on Pearson’s correlation were conducted for the global integration of nutritional composition, and the best score was compared for each species using the software PAST version 3.2, Oyvind Hammer, São Paulo, Brazil. The hierarchical cluster analyses were associated with heatmaps, in which raw data were log-transformed followed by correlation and cluster analyses.

## 3. Results and Discussion

### 3.1. Sample Collection and Species Identification

The beach-cast algae collected from the Brazilian northeast and southeast coasts exhibited great diversity in species: eight red macroalgae (Rhodophyta), three brown macroalgae (Phaeophyceae, Ochrophyta), and one green macroalgae (Chlorophyta) (Table S1).

### 3.2. Proximate Composition

The proximate composition is summarized in Table 1 and Supplementary Figure S1. Dry matter exhibited slight magnitude variation ranging from 89.0 ± 0.1% (*Halymenia brasiliana*) to 94.9 ± 0.3% (*Gracilaria domingensis*). The ash content of red beach-cast seaweeds ranged from 25.8 ± 0.2% to 58.3 ± 0.4%, while brown beach-cast seaweeds showed the lowest ash content of 6.5 ± 0.6% to 20.4 ± 0.8%. The ash content was highest in the green macroalga *Codium isthmocladum* (59.3 ± 1.5%) and in the red macroalga *Botryocladia occidentalis* (58.3 ± 0.4%). The ash content in seaweeds is high compared to plant vegetables. It includes macro-minerals and trace elements that show seasonal and environmental variation in the composition as described by Holdt and Kraan [16].

The protein content of macroalgae varies according to the species, environmental conditions, habitats, maturity, and seasonal differences, but are low in most brown seaweeds (three to 15%), moderate in green seaweeds (nine to 26%), and can attain 47% in red seaweeds [50,51,52,53]. The protein content varied within the species under study (Table 1, Figure S1) from 7.3 ± 0.1% to 19.7 ± 0.1% for red specimens, 10.9 ± 0.4% to 21.5 ± 0.2% for brown specimens, and lowest protein content of 5.1 ± 0.1% for the green alga *Codium isthmocladum*. It is worth noting that *Spatoglossum schroederi* attained 21.5 ± 0.2% protein content, an expressive protein amount for brown macroalgae. The protein content determined for the red and brown macroalgae in this study was similar to that found by other authors [11,18,51]. All these studies calculated the protein content taking 6.25 as a nitrogen-to-protein factor as done in our study for a better comparison. As described in the Materials and Methods section, we also used taxa-specific conversion factors for protein calculation and the data are shown in supplemental Table S2. 

Each seaweed class produces specific polysaccharides building the composition of the fibrillary and matrix-associated components of the cell wall as well as the storage carbohydrates. The amount of polysaccharides in seaweeds can reach up to 76% [16,54,55]. Tenorio et al. [10] determined a carbohydrate amount of 12% for red, 21% for brown, and 8% for green seaweeds. The analysis of beach-cast samples exhibited total carbohydrate content ranges from 31.4 ± 0.5% to 81.0 ± 0.7% (Table 1, Figure S1). The red seaweeds showed 31.4 ± 0.5% to 60.6 ± 0.3% of total carbohydrates, while total carbohydrates in brown seaweeds range from 59.1 ± 0.2% to 81.0 ± 0.7% and green macroalga *Codium isthmocladum* is 35.6 ± 1.5%. The total carbohydrate content of *Halymenia brasiliana* is comparable to that of the three *Halymenia* species from the Philippines (40.53–53.65%) as reported by Critchley et al. and Hurtado et al. [33,56]. The minimum number of soluble carbohydrates was determined to 5.3 ± 0.1 µg galactose/mg for *Codium isthmocladum*. The highest amount was found in *Dictyopteris jolyana* with 146.0 ± 0.1 µg galactose/mg (Table 1, Figure S1). *Gracilaria domingensis* with a soluble carbohydrate amount of 113.5 ± 0.1 µg galactose/mg is an agarophyte species with high agar yield and low agar strength explored in the Brazilian northeast as a source for the agar industry. These data suggested that red and brown beach-cast seaweeds from Brazil could be a good potential source of protein (respectively 19.7% and 21.5%) and carbohydrates (respectively 60.6% and 59.1%).

#### 3.2.1. Dietary Fibers

Seaweeds are rich in dietary fibers (>30%), particularly, in the soluble form, values that frequently exceed those of fruits and vegetables [11,17,57]. Tenorio et al. [10] determined the TDF amounts of 38% for red seaweeds, 36% for brown, and 38% for green seaweeds. Depending on the seaweed phyla, different types of dietary fibers exist. For red seaweeds (Rhodophyta), the soluble fibers are sulfated galactans (agar and carrageenans) or soluble xylans, which are components of the amorphous external cell wall matrix, such as small amounts of cellulose, xylans, galactans, hydroxyproline glycosides, mannans, and fucoidans depending on the taxa. For brown seaweeds (Phaeophyceae), the soluble fibers are alginates, fucans, and laminarans. Insoluble fibers are essentially composed of cellulose, except for some red alga, which consist of insoluble mannan and xylan [17,58,59]. The content of TDF in this study ranges from 22.1 ± 0.2 g/100 g to 65.8 ± 1.1 g/100 g (dry weight). In red seaweeds, TDF ranges from 25.0 ± 0.2 g/100 g to 46.8 ± 0.3 g/100 g. The brown seaweeds showed TDF amounts of 54.5 ± 2.3 g/100 g to 65.8 ± 1.1 g/100 g and *Codium isthmocladum* exhibited a TDF content of 22.1 ± 0.2 g/100 g. *Dictyopteris jolyana* and *Alsidium seaforthii* contained the highest TDF contents with 65.8 ± 1.1 g/100 g and 61.7 ± 4.9 g/100 g, of which 46.4 ± 1.0 g/100 g and 33.5 ± 0.6 g/100 g is SDF, while *Codium isthmocladum* had the lowest TDF content of 22.1 ± 0.2 g/100 g with 4.7 ± 0.5 g/100 g SDF. These results are consistent with other studies [17,56,60].

More than half of the investigated macroalgae have higher soluble fiber content than insoluble fiber content. *Spatoglossum schroederi*, *Zonaria tournefortii, Botryocladia occidentalis*, and *Codium isthmocladum* exhibited higher insoluble fiber content than soluble ones. The insoluble and soluble dietary fiber content (IDF and SDF) ranges between 5.1 ± 0.9 g/100 g to 46.2 ± 0.3 g/100 g and 4.7 ± 0.5 g/100 g to 46.4 ± 1.0 g/100 g, respectively (Table 1, Figure S1). Water-soluble and water-insoluble fibers have different physiological effects [5,19,61]. This high content of insoluble dietary fiber indicates a beneficial nutritional effect and thus the need to develop attractive fiber-based seaweed products.

#### 3.2.2. Mineral Composition

Seaweeds are known for their high mineral content, which is even 10–100 times higher than that of land vegetables [16,62,63]. This high content of minerals and trace elements is attributed to their ability to bind and accumulate inorganic components on the cell wall polysaccharides. [5,25,64]. Most macroalgae have high calcium, magnesium, potassium, sodium, and iron contents [12,63]. These elements were determined in all collected species (Table 2, Figure S2). A wide variation in mineral content was observed among the samples. High calcium content was observed in *Alsidium triquetrum* of 7.24 ± 0.01% and for *Alsidium seaforthii* 5.94 ± 0.05%. All red macroalgae contain an extraordinary level of potassium from 4.39 ± 0.01% for *Alsidium seaforthii* to 11.18 ± 0.06% for *Agardhiella ramosissima*, which is similar to the natural plasma level [12]. Notably, *Codium isthmocladum* possesses high magnesium content of 2.11 ± 0.01% and high sodium content of 14.90 ± 0.01%. The average content of iron followed the order Phaeophyceae (316.57 ± 1.88 ppm to 2306.33 ± 15.58 ppm) higher than Rhodophyta (112.72 ± 0.60 ppm to 1879.26 ± 24.36 ppm) and higher than Chlorophyta (310.40 ± 10.84 ppm). The level of minerals detected (Table 2) also fit within the ranges observed in previous reports in seaweeds [63]. Some of the analyzed seaweed species may be seen as good sources of calcium, potassium, magnesium, and iron. Cadmium and copper were not detected. Due to the high mineral content, algae could be a valuable addition to the dietary supplement. However, the linkage of certain minerals with anionic polysaccharides (alginate, agar, or carrageenan) might limit the absorption of these minerals [12].

#### 3.2.3. Free Amino Acids

Most seaweeds contain all essential amino acids at proportions comparable to traditional protein sources used for animal feed, such as soybean meal and fishmeal [2,5]. These amino acids occur as protein constituents and as free amino acids or salts of free amino acids. Free amino acids and peptides are key determinants in food taste, like L-glutamate, which is recognized for *Umami* taste and is rich in cheese, tomato, and kelps [65]. Other amino acids (alanine and glycine) also contribute to the distinct flavors of some marine algae [16].

As widely described, aspartic and glutamic acids constitute a large part of the amino acid fraction in seaweeds, while tryptophan is the first limiting amino acid in algae proteins. Generally, amino acid composition fluctuates seasonally as affected by environmental conditions and can also vary interspecifically [6,11,66,67]. The content, and the type of proteinaceous molecules, such as peptides and free amino acids, depends on several factors such as available light, salinity, temperature, wave force, nutrient and mineral availability, and carbohydrate levels [68].

Table 3 and Supplementary Figure S3 present the mean values from the analysis of free amino acid contents by HPLC. While aspartic acid (Asp), citrulline (Cit), glutamic acid (Glu), ornithine (Orn), serine (Ser), and tryptophan (Trp) are the most abundant free amino acids in brown seaweeds, red seaweeds possess a high amount of arginine (Arg), Asp, Cit, Glu, Orn, Ser, and Trp. The green species *Codium isthmocladum* showed a high amount of Arg, Asp, Cit, Glu, Leu, Ser, and Trp. The most significant observation pertains to the content of Cit that, for most species, is significantly higher than the contents of the other free amino acids. Citrulline is a common byproduct of other amino acids, such as Orn and Arg. Like other amino acids, they play many vital functional roles, such as the building of proteins, the synthesis of hormones, and neurotransmitters. The total free amino acids found in the beach-cast algae ranged from 290.4 ± 122.6 µg/g to 11,307.5 ± 4631.8 µg/g with a considerable wide variety of composition profiles and abundant characteristics for each material. Therefore, considering the functional role of free amino acids, immediate availability, and possible seaweed supplementation as a natural source, some species studied here can be proposed as natural amino acid stock.

#### 3.2.4. Fatty Acids

Lipids represent up to 4% of the seaweed, and relatively low content of saturated fatty acids, as well as a substantial amount of PUFA, as compared to land vegetables [56]. Lipid levels and composition, including fatty acid profiles, vary according to a taxonomic entity, season, geographic regions, and growth conditions [56]. Marine lipids consist of a substantial number of long-chain PUFAs, with n-3 fatty acids as the significant component and mono-unsaturated fatty acids [16]. PUFAs greater than C18 are abundantly found in marine species, with green algae being rich in C18 PUFAs (ALA, STA, and LA) and red algae being rich in C20 PUFAs (AA and EPA), while brown algae exhibit both appreciable amounts [5,69,70].

The fatty acid composition of the seaweeds under study is shown in Table 4 and Figure S4. In all analyzed seaweeds, palmitic acid (C16:0) was the single most abundant saturated fatty acid. The content of C16:0 was highest in *Agardhiella ramosissima* with 62.77 ± 10.63 g/100 g, and lower levels were found in *Dictyopteris jolyana* with 21.05 ± 1.05 g/100 g. Furthermore, the macroalga varieties had minor levels of myristic acid (C14:0) ranging from 1.92 ± 0.21 g/100 g to 14.01 ± 0.77 g/100 g. In agreement with other studies, the most abundant fatty acid in the algae, apart from C16:0, was C18:1, which was not detected in *Agardhiella ramosissima* [31,71,72]. Eight seaweeds also contained the essential fatty acids C18:2 (linoleic acid) and C18:3 (linolenic acid). Since humans are incapable of synthesizing PUFAs with more than 18 atoms of carbon, n-3 PUFAs are of nutritional importance and must be added as a dietary supplement or as part of a balanced diet [72].

### 3.3. Protein Solubility

Macroalgae have a robust polysaccharide-rich cell wall, and the cell wall mucilage reduces protein extractability. The extractability of proteins is influenced by both the ionic interactions between the cell wall and the proteins as well as the high viscosity exerted via the polysaccharides in a water solution [73]. Hence, the pH had a significant influence on the solubility of the seaweed proteins. Several studies have shown that the extractability and recovery of seaweed proteins could be increased with the pH-shift process, using alkaline protein solubilization followed by isoelectric precipitation, an efficient way to produce extracts with high protein concentrations [53].

The protein solubility in water at pH values between pH 2 and pH 13 was measured (Table 5). The protein solubility increased with increasing pH in all species. This was also reported by Harrysson et al. [71] and Vilg et al. [74] for *Porphyra* sp., *Ulva* sp., *Saccharina* sp. The maximum solubility of 54.8 ± 1.8% and 52.5 ± 1.5% was achieved at pH 13 for *Alsidium seaforthii* and *Gracilaria domingensis*, respectively (Table 4, Figure S5). Both red algae species also exhibited high protein contents of 19.7% and 16.8%, respectively. Lowest values for solubility at pH 13 were observed for *Dictyopteris jolyana* (25.3%), *Spatoglossum schroederi* (26.3%), and *Spyridia clavata* (26.0%). The brown algae *Spatoglossum schroederi* also showed a comparative high protein content of 21.5 ± 0.2%, but a very low protein solubility of 26.3 ± 1.3% at pH 13. The solubility decreased with declining pH and finally reached a plateau at pH 6–8 with a maximum protein solubility of 35% up to 42% for some studied algae. The protein solubility curve of macroalgae differs from that of legumes, whose solubility curves show a minimum at pH 4–6 (isoelectric point) [75]. These differences result from the type of protein extracted. Legume proteins are storage proteins, whereas alga proteins are mainly structural proteins, enzymes, or chromoproteins, which result in different solubility properties [74].

### 3.4. Integrated Cluster Analyses and Heatmaps

**Figure 1:**
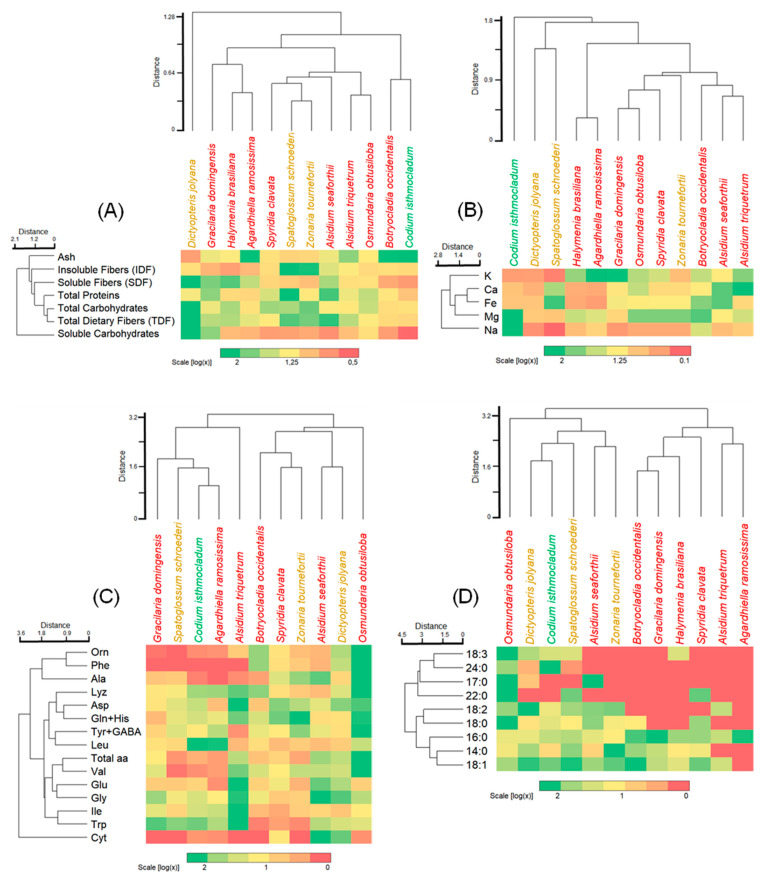
Heatmap of the biplot hierarchical Euclidean cluster analysis from beach-cast macroalgae for (**A**) proximate composition; (**B**) macro (Ca, K, Mg, and Na) and micro (Fe) elements; (**C**) free amino acid profile (amino acid values < 0.5 are represented as zero); (**D**) fatty acids composition (not detected fatty acids are represented as zero). Values represent the log transformation.

Biplot hierarchical Euclidean clusters were conducted on the basis of subgroups of chemical characterization associated with heatmaps: proximate composition (Figure 1A), macro (Ca, K, Mg, and Na) and micro (Fe) elements (Figure 1B), amino acid profile (Figure 1C), and fatty acid composition (Figure 1D).

From the proximate composition, three subclusters were identified (Figure 1A):

(a) an isolated set by the brown alga *Dictyopteris jolyana* with high amounts of TDF, SDF, and carbohydrates, and moderate amounts of IDF and proteins;
(b) a set including red and brown algae with variable number of parameters from high, moderate, and low;
(c) a set comprising the red alga *Botryocladia occidentalis* and green alga *Codium isthmocladum* with moderate to low values, except for ash content.

Large amounts of TDF, especially the IDF, are a preferable feature as health-promoting benefits for gut bacteria [76], in which almost all beach-cast seaweeds from this study exhibited high to moderate contents. The fiber fraction from the beach-cast material contain polysaccharides of high molecular weight that are insoluble in water, except in hot water [16]. They are not found as free carbohydrates, since the soluble carbohydrate content was low. This trait is an interesting advantage, as these fibers can be available after passing through the digestive tract or through specific treatments for their extraction. Therefore, all species studied are profitable sources of dietary fibers, except for *Codium isthmocladum,* which showed higher ash content associated with an elevated number of minerals, suitable for different applications than dietary fiber sources.

A high mineral content is a characteristic of many seaweeds [16,62,63]. From the cluster analysis (Figure 1B), a wide variation was observed for macro (Ca, K, Mg, Na) and micro elements (Fe). A notable observation is the high concentration of magnesium and sodium in *Codium isthmocladum*, which is of nutritional concern if consumed in excess. All other beach-cast algae, however, possess lower levels of sodium. Other macro and microelements can be appreciated from sources of marine algae as described in our results.

As the mineral cluster, the beach-cast seaweeds exhibited a broad variation in free amino acids (Figure 1C). From the cluster, we can identify four major sets:

(a) one with relatively small amounts of amino acids, such as Gracilaria domingensis, Spatoglossum schoroederi, Codium isthmocladum, and Agardhiella ramosissima;
(b) an alone set by Alsidium triquetrum with high levels of half of the amino acids;
(c) a major set with moderate to a high concentration of almost all amino acids, and
(d) an alone set by Osmundaria obstusiloba exhibiting high levels of almost all amino acids.

This wide amino acid profile is an important feature for the natural ingredient industries. Amino acids like L-glutamate are determinants in Umami taste, typically of Asian cuisine. An important highlight of the amino acid profile from this study represents the quantification of free amino acids; therefore, their immediate availability can make the valorization of beach-cast seaweed supplementation as a natural source of amino acids possible.

Seaweeds are not commonly seen as a source of lipids or fatty acids, since lipids represent only up to 4% of the dry matter. Nonetheless, despite their small amounts, they are seen as potential sources of certain fatty acids, especially PUFAs that humans can not synthesize. PUFAs with 18 carbon atoms are of nutritional importance and must be added as a diet, in which the beach-cast seaweeds showed relevance in these fatty acids (Figure 1D), with species exhibiting a large amount of almost all fatty acids and others with high concentrations of half of the fatty acids.

## 4. Conclusions

The data from this study did not show a clear pattern between the phyla and the composition of the individual nutrients. Interesting nutritional profiles were highlighted for *Spatoglossum schroederi* and *Alsidium seaforthii* with appreciable protein contents of 21.5 ± 0.2%, 19.7 ± 0.1%, respectively, and high amounts of total dietary fiber of 59.2 ± 0.4%, 61.7 ± 4.9%, respectively, and low ash, and low soluble carbohydrate content. *Dictyopteris jolyana* revealed the highest amount of TDF (65.8 ± 1.1%), SDF (46.4 ± 1.0%), and total and soluble carbohydrates of 81.0 ± 0.7%, 146.0 ± 0.1 µg galactose/mg on dry mass basis, respectively, compared to the other studied species. The free amino acid composition was highest for *Alsidium triquetrum* and *Osmundaria obtusiloba*. Additionally, *Osmundaria obtusiloba* exhibited high levels of PUFA. These algae are promising for aquaculture cultivation to provide valuable raw materials for future production of functional ingredients for the food industry.

The controlled cultivation of beach-cast algae species has huge potential to contribute to a sustainable, environmentally-friendly local marine industry. One of the main challenges in realizing this vision is the development of controlled growing conditions for these algae species in aquaculture. For the extraction of individual compounds as food ingredients, more cost-effective extraction and isolation/concentration methods need to be developed. In addition, undesirable components such as off-flavors and colorants must be removed to increase their sensory perceptions and usability for the food industry.

## Figures and Tables

**Table 1:** Proximate composition of beach-cast macroalgae (g/100 g = % and * µg galactose/mg on dry mass basis). Values represent the average of three replicates (mean ± SD), and letters indicate statistical significance (*p* < 0.05). Total carbohydrates calculated by difference = 100-total protein-ash.

Species	Dry Matter	Ash	Total Dietary Fibers (TDF)	Soluble Fibers (SDF)	Insoluble Fibers (IDF)	SDF/TDF	IDF/TDF	Total Proteins	Total Carbohydrates	Soluble Carbohydrates *
**Rhodophyta (red algae)**										
Agardhiella ramosissima	92.2 ± 0.2 ^cd^	57.3 ± 0.2 ^a^	40.1 ± 1.5 ^cd^	31.5 ± 2.1 ^d^	8.6 ± 0.7 ^d^	0.79	0.21	7.3 ± 0.1 ^i^	35.4 ± 0.3 ^i^	25.9 ± 0.1 ^de^
Alsidium seaforthii	94.5 ± 0.3 ^a^	34.7 ± 1.0 ^c^	61.7 ± 4.9 ^a^	33.5 ± 0.6 ^d^	28.2 ± 4.7 ^b^	0.54	0.46	19.7 ± 0.1 ^b^	45.6 ± 0.9 ^g^	11.9 ± 0.1 ^de^
Alsidium triquetrum	92.7 ± 0.1 ^bc^	46.5 ± 0.4 ^b^	45.2 ± 4.8 ^c^	19.3 ± 2.4 ^e^	25.9 ± 2.3 ^b^	0.43	0.57	12.8 ± 0.2 ^f^	40.7 ± 0.5 ^h^	32.3 ± 0.1 ^d^
Botryocladia occidentalis	92.7 ± 0.1 ^bc^	58.3 ± 0.4 ^a^	25.0 ± 0.2 ^e^	6.2 ± 0.4 ^h^	18.8 ± 0.6 ^c^	0.25	0.75	10.3 ± 0.2 ^g^	31.4 ± 0.5 ^j^	15.8 ± 0.1 ^de^
Gracilaria domingensis	94.9 ± 0.3 ^a^	35.2 ± 0.9 ^c^	45.9 ± 0.8 ^c^	37.5 ± 0.9 ^c^	8.3 ± 0.1 ^d^	0.82	0.18	16.8 ± 0.1 ^c^	47.9 ± 0.8 ^f^	113.5 ± 0.1 ^b^
Halymenia brasiliana	89.0 ± 0.1 ^h^	33.7 ± 1.0 ^c^	46.8 ± 0.3 ^c^	41.8 ± 1.1 ^b^	5.1 ± 0.9 ^d^	0.89	0.11	8.2 ± 0.4 ^h^	58.1 ± 1.4 ^d^	30.0 ± 0.1 ^d^
Osmundaria obtusiloba	92.9 ± 0.1 ^b^	31.2 ± 0.1 ^d^	36.9 ± 3.5 ^d^	19.8 ± 1.7 ^e^	17.1 ± 1.7 ^c^	0.54	0.46	14.6 ± 0.2 ^d^	54.1 ± 0.2 ^e^	58.9 ± 0.1 ^c^
Spyridia clavata	90.8 ± 0.2 ^g^	25.8 ± 0.2 ^e^	33.7 ± 0.2 ^d^	16.0 ± 0.9 ^ef^	17.8 ± 0.9 ^c^	0.47	0.53	13.6 ± 0.2 ^e^	60.6 ± 0.3 ^c^	15.8 ± 0.1 ^cde^
**Phaeophyceae (brown algae)**										
Dictyopteris jolyana	92.0 ± 0.2 ^de^	6.5 ± 0.6 ^g^	65.8 ± 1.1 ^a^	46.4 ± 1.0 ^a^	19.4 ± 0.2 ^c^	0.71	0.29	12.5 ± 0.2 ^f^	81.0 ± 0.7 ^a^	146.0 ± 0.1 ^a^
Spatoglossum schroederi	91.3 ± 0.4 ^fg^	19.4 ± 0.4 ^f^	59.2 ± 0.4 ^ab^	13.1 ± 0.3 ^fg^	46.2 ± 0.3 ^a^	0.22	0.78	21.5 ± 0.2 ^a^	59.1 ± 0.2 ^cd^	16.6 ± 0.1 ^de^
Zonaria tournefortii	91.6 ± 0.4 ^ef^	20.4 ± 0.8 ^f^	54.5 ± 2.3 ^b^	10.2 ± 1.8 ^g^	44.3 ± 1.1 ^a^	0.19	0.81	10.9 ± 0.4 ^g^	68.7 ± 0.5 ^b^	19.5 ± 0.1 ^de^
**Chlorophyta (green algae)**										
Codium isthmocladum	93.1 ± 0.1 ^b^	59.3 ± 1.5 ^a^	22.1 ± 0.2 ^e^	4.7 ± 0.5 ^h^	17.4 ± 0.3 ^c^	0.21	0.79	5.1 ± 0.1 ^j^	35.6 ± 1.5 ^i^	5.3 ± 0.1 ^e^

**Table 2:** Macro (N, P, Ca, K, Mg, and Na) and micro (Fe) elements as well as trace metals of beach-cast macroalgae (g/100 g = % and * ppm on dry mass basis). Values represent the average of three replicates (mean ± SD), and letters indicate statistical significance (*p* < 0.05). nd = not detected.

Species	Ca	K	Mg	Na	Fe *	Cd	Cu
**Rhodophyta (red algae)**							
Agardhiella ramosissima	0.50 ± 0.01 ^i^	11.18 ± 0.06 ^a^	0.96 ± 0.01 ^f^	2.83 ± 0.01 ^c^	112.72 ± 0.60 ^j^	nd	nd
Alsidium seaforthii	5.94 ± 0.05 ^b^	4.39 ± 0.01 ^h^	0.81 ± 0.01 ^h^	1.72 ± 0.01 ^e^	1879.26 ± 24.36 ^c^	nd	nd
Alsidium triquetrum	7.24 ± 0.01 ^a^	7.76 ± 0.03 ^c^	1.03 ± 0.01 ^e^	2.44 ± 0.01 ^d^	509.17 ± 2.99 ^h^	nd	nd
Botryocladia occidentalis	2.82 ± 0.03 ^e^	5.90 ± 0.01 ^f^	1.74 ± 0.01 ^b^	7.17 ± 0.06 ^b^	1613.59 ± 12.54 ^d^	nd	nd
Gracilaria domingensis	2.29 ± 0.03 ^f^	10.80 ± 0.01 ^b^	0.45 ± 0.01 ^i^	0.65 ± 0.01 ^i^	941.38 ± 14.01 ^e^	nd	nd
Halymenia brasiliana	0.54 ± 0.01 ^i^	7.24 ± 0.03 ^d^	1.12 ± 0.02 ^d^	1.62 ± 0.06 ^f^	153.21 ± 0.49 ^j^	nd	nd
Osmundaria obtusiloba	3.84 ± 0.03 ^d^	6.25 ± 0.02 ^e^	0.44 ± 0.01 ^i^	0.29 ± 0.01 ^k^	832.60 ± 2.24 ^g^	nd	nd
Spyridia clavata	1.70 ± 0.03 ^g^	5.43 ± 0.01 ^g^	1.51 ± 0.01 ^c^	1.04 ± 0.01 ^g^	878.81 ± 14.26 ^f^	nd	nd
**Phaeophyceae (brown algae)**							
Dictyopteris jolyana	0.58 ± 0.01 ^i^	0.56 ± 0.01 ^j^	0.33 ± 0.01 ^j^	0.54 ± 0.01 ^j^	316.57 ± 1.88 ^i^	nd	nd
Spatoglossum schroederi	4.31 ± 0.05 ^c^	0.26 ± 0.01 ^k^	0.30 ± 0.01 ^k^	0.15 ± 0.01 ^l^	2021.13 ± 28.13 ^b^	nd	nd
Zonaria tournefortii	2.75 ± 0.01 ^e^	1.28 ± 0.01 ^i^	0.86 ± 0.01 ^g^	0.81 ± 0.01 ^h^	2306.33 ± 15.58 ^a^	nd	nd
**Chlorophyta (green algae)**							
Codium isthmocladum	0.93 ± 0.01 ^h^	0.54 ± 0.01 ^j^	2.11 ± 0.01 ^a^	14.90 ± 0.01 ^a^	310.40 ± 10.84 ^i^	nd	nd

**Table 3:** Free amino acid composition of beach-cast macroalgae (µg/g on dry mass basis). Values represent the average of three replicates (mean ± SD), and letters indicate the statistical significance (*p* < 0.05).

Species	Ala	Arg	Asn	Asp	Cit	Gln + His	Glu	Gly	Ile	Leu
**Rhodophyta (red algae)**										
Agardhiella ramosissima	0.4 ± 0.1 ^d^	15.4 ± 0.3 ^c^	8.5 ± 0.8 ^b^	12.0 ± 0.8 ^g^	65.8 ± 1.7 ^d^	14.5 ± 0.2 ^c^	11.8 ± 1.1 ^e^	3.2 ± 0.2 ^c^	6.8 ± 0.3 ^b^	30.3 ± 0.2 ^b^
Alsidium seaforthii	36.8 ± 14.9 ^b^	4.9 ± 1.5 ^c^	35.7 ± 0.1 ^b^	42.7 ± 6.4 ^ef^	2878.4 ± 211.5 ^a^	8.2 ± 3.3 ^c^	327.1 ± 44.2 ^b^	19.0 ± 7.9 ^a^	2.4 ± 0.5 ^cd^	2.1 ± 0.6 ^de^
Alsidium triquetrum	1.2 ± 0.4 ^d^	1682.6 ± 76.7 ^a^	2.6 ± 1.1 ^b^	190.8 ± 5.5 ^a^	0.9 ± 0.5 ^d^	41.1 ± 4.7 ^b^	513.4 ± 9.9 ^a^	16.0 ± 1.7 ^a^	23.7 ± 2.0 ^a^	1.8 ± 0.4 ^e^
Botryocladia occidentalis	1.9 ± 0.4 ^d^	2.2 ± 0.4 ^c^	6.3 ± 0.2 ^b^	13.9 ± 1.5 ^g^	9.1 ± 0.9 ^d^	17.6 ± 3.3 ^c^	46.3 ± 3.4 ^e^	3.9 ± 0.6 ^c^	1.4 ± 0.3 ^d^	4.0 ± 0.6 ^de^
Gracilaria domingensis	4.0 ± 1.2 ^d^	293.0 ± 78.7 ^b^	4.4 ± 0.9 ^b^	26.3 ± 5.7 ^fg^	8.5 ± 1.6 ^d^	3.9 ± 0.6 ^c^	42.7 ± 9.7 ^e^	8.1 ± 1.9 ^bc^	5.3 ± 0.9 ^bc^	4.3 ± 1.5 ^d^
Osmundaria obtusiloba	56.1 ± 5.7 ^a^	2.1 ± 0.2 ^c^	6788.1 ± 751.8 ^a^	139.8 ± 17.4 ^b^	67.4 ± 15.2 ^d^	59.7 ± 8.3 ^ab^	45.8 ± 8.6 ^e^	6.1 ± 1.3 ^bc^	4.1 ± 0.1 ^bcd^	9.9 ± 0.7 ^c^
Spyridia clavata	13.6 ± 3.6 ^cd^	2.0 ± 0.5 ^c^	5.9 ± 0.6 ^b^	96.8 ± 5.4 ^c^	416.4 ± 16.7 ^c^	40.8 ± 1.1 ^b^	103.3 ± 3.1 ^d^	1.5 ± 1.9 ^c^	0.9 ± 0.2 ^d^	2.0 ± 0.6 ^de^
**Phaeophyceae (brown algae)**										
Dictyopteris jolyana	5.8 ± 0.6 ^d^	2.4 ± 0.01 ^c^	51.7 ± 1.0 ^b^	96.0 ± 3.2 ^c^	1832.7 ± 0.1 ^b^	8.8 ± 0.01 ^c^	184.8 ± 0.1 ^c^	13.6 ± 0.01 ^ab^	1.7 ± 0.2 ^d^	2.8 ± 1.3 ^de^
Spatoglossum schroederi	4.6 ± 0.1 ^d^	1.3 ± 0.4 ^c^	5.0 ± 0.3 ^b^	44.6 ± 0.1 ^ef^	9.0 ± 4.5 ^d^	15.0 ± 0.01 ^c^	41.2 ± 0.1 ^e^	3.7 ± 0.9 ^c^	2.3 ± 1.5 ^cd^	3.6 ± 0.7 ^de^
Zonaria tournefortii	24.9 ± 5.7 ^bc^	1.0 ± 0.3 ^c^	4.9 ± 2.0 ^b^	66.4 ± 0.1 ^d^	24.2 ± 0.1 ^d^	69.0 ± 20.6 ^a^	24.5 ± 0.1 ^e^	1.7 ± 0.4 ^c^	2.0 ± 0.8 ^cd^	2.8 ± 1.2 ^de^
**Chlorophyta (green algae)**										
Codium isthmocladum	0.9 ± 0.2 ^d^	20.7 ± 0.1 ^c^	5.9 ± 2.3 ^b^	55.8 ± 2.8 ^de^	78.0 ± 0.1 ^d^	18.2 ± 0.9 ^c^	49.6 ± 0.5 ^e^	4.8 ± 1.2 ^c^	6.3 ± 2.5 ^b^	33.7 ± 0.1 ^a^
**Species**		**Lys**	**Met**	**Orn**	**Phe**	**Ser**	**Thr**	**Trp**	**Tyr + GABA**	**Val**
**Rhodophyta (red algae)**										
Agardhiella ramosissima		15.7 ± 2.7 ^bcd^	0.8 ± 0.2 ^b^	19.8 ± 1.9 ^e^	1.3 ± 0.2 ^de^	46.7 ± 1.6 ^cde^	1.1 ± 0.1 ^bc^	45.8 ± 1.6 ^cde^	2.7 ± 0.3 ^b^	0.7 ± 0.3 ^e^
Alsidium seaforthii		2.2 ± 0.6 ^f^	1.0 ± 0.2 ^b^	42.8 ± 3.5 ^de^	7.6 ± 0.4 ^de^	43.9 ± 9.1 ^cde^	nd	43.9 ± 9.1 ^cde^	1.8 ± 0.1 ^b^	6.7 ± 1.3 ^c^
Alsidium triquetrum		24.6 ± 1.1 ^b^	3.3 ± 1.3 ^b^	67.3 ± 3.9 ^de^	1.0 ± 0.1 ^de^	1128.3 ± 5.2 ^a^	10.1 ± 5.5 ^a^	128.3 ± 5.2 ^a^	0.2 ± 0.1 ^b^	13.0 ± 1.4 ^b^
Botryocladia occidentalis		18.0 ± 2.9 ^bc^	0.5 ± 0.2 ^b^	361.7 ± 27.2 ^b^	70.2 ± 9.2 ^b^	2.1 ± 0.5 ^g^	0.2 ± 0.1 ^c^	2.1 ± 0.5 ^f^	1.8 ± 0.5 ^b^	5.8 ± 1.5 ^cd^
Gracilaria domingensis		5.4 ± 0.6 ^ef^	732.3 ± 212.7 ^a^	10.8 ± 2.1 ^e^	0.5 ± 0.1 ^de^	91.6 ± 34.6 ^b^	0.9 ± 0.6 ^bc^	91.6 ± 34.6 ^b^	0.8 ± 0.1 ^b^	5.1 ± 1.5 ^cd^
Osmundaria obtusiloba		37.9 ± 8.3 ^a^	1.2 ± 0.2 ^b^	814.6 ± 68.2 ^a^	156.2 ± 17.0 ^a^	29.9 ± 4.9 ^ef^	5.6 ± 1.3 ^ab^	29.9 ± 4.9 ^ef^	8.4 ± 1.6 ^a^	22.4 ± 1.0 ^a^
Spyridia clavata		4.6 ± 1.4 ^f^	0.5 ± 0.2 ^b^	108.7 ± 0.9 ^d^	18.6 ± 3.9 ^d^	4.2 ± 0.4 ^g^	0.7 ± 0.4 ^bc^	4.2 ± 0.4 ^f^	1.5 ± 0.2 ^b^	3.6 ± 0.6 ^d^
**Phaeophyceae (brown algae)**										
Dictyopteris jolyana		9.0 ± 0.5 ^cdef^	0.7 ± 0.1 ^b^	289.5 ± 21.4 ^c^	46.8 ± 5.6 ^c^	32.7 ± 5.0 ^def^	1.9 ± 0.1 ^bc^	32.7 ± 5.0 ^def^	1.0 ± 0.1 ^b^	6.0 ± 0.7 ^cd^
Spatoglossum schroederi		7.1 ± 4.3 ^def^	1.3 ± 0.7 ^b^	7.3 ± 5.1 ^e^	0.4 ± 0.3 ^e^	64.5 ± 0.0 ^bcd^	0.4 ± 0.3 ^c^	64.5 ± 0.1 ^bcd^	2.0 ± 2.5 ^b^	0.3 ± 0.1 ^e^
Zonaria tournefortii		3.1 ± 0.8 ^f^	0.2 ± 0.1 ^b^	50.9 ± 15.9 ^de^	12.7 ± 0.6 ^de^	2.6 ± 1.2 ^g^	1.2 ± 0.2 ^bc^	2.6 ± 1.2 ^f^	4.1 ± 0.5 ^b^	6.7 ± 0.1 ^c^
**Chlorophyta (green algae)**										
Codium isthmocladum		15.0 ± 4.8 ^bcde^	3.6 ± 3.3 ^b^	17.6 ± 8.5 ^e^	0.7 ± 0.7 ^de^	74.8 ± 5.3 ^bc^	1.0 ± 0.5 ^bc^	74.8 ± 5.3 ^bc^	1.3 ± 0.8 ^b^	0.5 ± 0.4 ^e^

**Table 4:** Fatty acid composition of beach-cast macroalgae (g/100 g on dry mass basis). Values represent the average of three replicates (mean ± SD), and letters indicate statistical significance (*p* < 0.05). Statistical analysis was performed only for amounts over 10. nd = not detected.

Species	14:0	16:0	17:0	18:0	22:0	24:0	18:1	18:2	18:3
**Rhodophyta (red algae)**									
Agardhiella ramosissima	nd	62.77 ± 10.63 ^a^	nd	nd	nd	nd	nd	nd	nd
Alsidium seaforthii	1.92 ± 0.21 ^g^	21.65 ± 0.47 ^d^	3.56 ± 1.43	2.63 ± 0.20	nd	nd	10.40 ± 0.87 ^bcd^	4.68 ± 1.05	nd
Alsidium triquetrum	nd	27.36 ± 0.33 ^cd^	nd	nd	nd	nd	7.50 ± 0.31 ^d^	3.24 ± 0.10	nd
Botryocladia occidentalis	7.32 ± 0.08 ^bc^	53.58 ± 0.78 ^b^	nd	1.11 ± 1.93	nd	nd	20.09 ± 0.63 ^a^	nd	nd
Gracilaria domingensis	5.53 ± 0.19 ^d^	68.16 ± 1.15 ^a^	nd	nd	nd	nd	8.38 ± 0.10 ^cd^	nd	nd
Halymenia brasiliana	3.32 ± 0.13 ^f^	35.32 ± 1.08 ^c^	nd	nd	nd	nd	6.62 ± 0.34 ^d^	nd	2.01 ± 1.74
Osmundaria obtusiloba	4.11 ± 0.64 ^e^	21.27 ± 2.50 ^d^	3.52 ± 1.50	4.67 ± 1.05	5.18 ± 2.04	3.14 ± 1.63	9.82 ± 1.69 ^cd^	4.21 ± 1.41	5.86 ± 0.48
Spyridia clavata	4.13 ± 0.32 ^e^	35.50 ± 3.81 ^c^	nd	2.45 ± 2.27	3.64 ± 0.00	nd	13.99 ± 1.45 ^abcd^	nd	nd
**Phaeophyceae (brown algae)**									
Dictyopteris jolyana	6.89 ± 0.35 ^c^	21.05 ± 1.05 ^d^	0.34 ± 0.58	0.91 ± 0.05	nd	0.37 ± 0.63	17.67 ± 0.90 ^bcd^	7.89 ± 0.40	2.69 ± 0.13
Spatoglossum schroederi	7.86 ± 0.25 ^b^	30.44 ± 1.19 ^c^	nd	1.16 ± 0.25	3.54 ± 0.00	0.19 ± 0.16	18.26 ± 0.86 ^ab^	3.93 ± 0.18	2.01 ± 0.06
Zonaria tournefortii	14.01 ± 0.77 ^a^	22.49 ± 1.17 ^d^	0.44 ± 0.75	1.69 ± 0.09	nd	nd	15.58 ± 1.24 ^abc^	5.40 ± 0.39	nd
**Chlorophyta (green algae)**									
Codium isthmocladum	2.20 ± 0.18 ^g^	28.03 ± 2.67 ^cd^	nd	1.50 ± 0.12	nd	6.09 ± 0.69	13.66 ± 1.35 ^abcd^	2.36 ± 0.18	2.10 ± 0.19

**Table 5:** Solubility of total proteins from beach-cast macroalgae (%) at different pH levels. Values represent the average of three replicates (mean ± SD), and letters indicate statistical significance (*p* < 0.05). nd = not detected.

Species	pH 2	pH 4	pH 6	pH 8	pH 10	pH 12	pH 13	Total Proteins
**Rhodophyta (red algae)**								
Agardhiella ramosissima	27.3 ± 2.5 ^c^	22.4 ± 2.5 ^cde^	34.7 ± 0.1 ^b^	40.9 ± 8.7 ^a^	33.5 ± 1.3 ^cd^	29.8 ± 2.5 ^d^	51.0 ± 1.2 ^a^	7.3 ± 0.1 ^i^
Alsidium seaforthii	27.4 ± 4.0 ^c^	39.5 ± 0.9 ^a^	40.4 ± 0.9 ^a^	38.6 ± 0.9 ^a^	40.8 ± 2.2 ^a^	42.2 ± 0.9 ^b^	54.8 ± 1.8 ^a^	19.7 ± 0.1 ^b^
Alsidium triquetrum	23.7 ± 0.01 ^cd^	25.9 ± 2.1 ^cd^	25.9 ± 0.7 ^c^	27.3 ± 0.7 ^b^	29.3 ± 0.1 ^cd^	35.6 ± 0.6 ^c^	nd	12.8 ± 0.2 ^f^
Gracilaria domingensis	32.8 ± 0.5 ^b^	33.9 ± 0.5 ^a^	37.4 ± 0.1 ^ab^	35.9 ± 0.5 ^a^	38.0 ± 1.6 ^cd^	38.5 ± 0.1 ^bc^	52.5 ± 1.5 ^a^	16.8 ± 0.1 ^c^
Halymenia brasiliana	18.2 ± 0.1 ^e^	19.4 ± 1.1 ^de^	22.7 ± 0.1 ^cd^	19.3 ± 1.1 ^cd^	20.5 ± 2.3 ^e^	21.6 ± 1.1 ^e^	34.0 ± 2.3 ^bc^	8.2 ± 0.4 ^h^
Osmundaria obtusiloba	39.8 ± 1.6 ^a^	37.2 ± 2.1 ^a^	35.0 ± 3.2 ^b^	39.9 ± 0.5 ^a^	32.4 ± 2.7 ^cd^	33.4 ± 3.7 ^cd^	34.0 ± 0.1 ^bc^	14.6 ± 0.2 ^d^
Spyridia clavata	27.2 ± 0.7 ^c^	33.2 ± 0.1 ^ab^	42.0 ± 3.3 ^a^	37.2 ± 1.3 ^a^	35.2 ± 3.3 ^abc^	48.6 ± 2.0 ^a^	26.0 ± 0.8 ^c^	13.6 ± 0.2 ^e^
**Phaeophyceae (brown algae)**								
Dictyopteris jolyana	3.6 ± 0.7 ^fg^	4.3 ± 0.1 ^f^	6.5 ± 0.7 ^ef^	7.3 ± 0.1 ^ef^	6.5 ± 0.7 ^f^	7.2 ± 0.1 ^f^	25.3 ± 0.8 ^c^	12.5 ± 0.2 ^f^
Spatoglossum schroederi	0.8 ± 0.1 ^g^	nd	2.9 ± 2.1 ^f^	4.2 ± 0.1 ^ef^	5.8 ± 0.8 ^f^	6.3 ± 0.4 ^f^	26.3 ± 1.3 ^c^	21.5 ± 0.2 ^a^
Zonaria tournefortii	6.7 ± 0.1 ^f^	15.9 ± 5.9 ^e^	9.2 ± 0.8 ^e^	11.7 ± 1.7 ^de^	24.2 ± 2.5 ^de^	29.2 ± 2.5 ^d^	40.0 ± 6.7 ^b^	10.9 ± 0.4 ^g^
**Chlorophyta (green algae)**								
Codium isthmocladum	nd	1.8 ± 1.8 ^f^	3.5 ± 0.1 ^f^	1.8 ± 1.8 ^f^	3.5 ± 0.1 ^f^	7.0 ± 0.1 ^f^	nd	5.1 ± 0.1 ^j^

## Supplementary Materials

The following are available online at https://www.mdpi.com/article/10.3390/foods11091201/s1, Table S1. Summary of the beach-cast algae collected from southeast and northeast beaches, Brazil. CE: Ceará State, northeast coast. ES: Espírito Santo State, southeast coast. PE: Pernambuco State, northeast coast. Table S2: Proximate composition of beach-cast macroalgae (g/100 g = % and * µg galactose/mg on dry mass basis). Values represent the average of three replicates (mean ± SD) and letters indicate the statistical significance (*p* < 0.05). Total carbohydrates calculated by difference = 100-total protein-ash. Figure S1. Proximate composition of beach-cast macroalgae. Values represent the average of three replicates (mean ± SD), and letters indicate the statistical significance (*p* < 0.05). Figure S2. Macro (N, P, Ca, K, Mg, and Na) and micro (Fe) elements as well as trace metals of beach-cast macroalgae. Values represent the average of three technical replicates (mean ± SD), and letters indicate the statistical significance (*p* < 0.05). Figure S3. Free amino acids composition of beach-cast macroalgae. Values represent the average of three replicates (mean ± SD), and letters indicate the statistical significance (*p* < 0.05). Figure S4. Fatty acids composition of beach-cast macroalgae. Values represent the average of three replicates (mean ± SD), and letters indicate the statistical significance (p < 0.05). Statistical analysis was conducted only for amounts over 10. Figure S5. Solubility of total proteins from beach-cast macroalgae at different pH levels. Values represent the average of three replicates (mean ± SD), and letters indicate the statistical significance (*p* < 0.05).

## References

[1] Angell A.R., Angell S.F., de Nys R., Paul N.A. (2016). Seaweed as a protein source for mono-gastric livestock. Trends Food Sci. Technol..

[2] Angell A.R., Mata L., de Nys R., Paul N.A. (2016). The protein content of seaweeds: A universal nitrogen-to-protein conversion factor of five. J. Appl. Phycol..

[3] Bolton J.J., Robertson-Andersson D.V., Shuuluka D., Kandjengo L. (2009). Growing *Ulva* (chlorophyta) in integrated systems as a commercial crop for abalone feed in South Africa: A swot analysis. J. Appl. Phycol..

[4] Mata L., Magnusson M., Paul N.A., de Nys R. (2016). The intensive land-based production of the green seaweeds *Derbesia tenuissima* and *Ulva ohnoi*: Biomass and bioproducts. J. Appl. Phycol..

[5] Domínguez H. (2013). 1-Algae as a source of biologically active ingredients for the formulation of functional foods and nutraceuticals. Functional Ingredients from Algae for Foods and Nutraceuticals.

[6] Fleurence J. (1999). Seaweed proteins: Biochemical, nutritional aspects and potential uses. Trends Food Sci. Technol..

[7] Fleurence J. (1999). The enzymatic degradation of algal cell walls: A useful approach for improving protein accessibility?. J. Appl. Phycol..

[8] Fleurence J., Le Coeur C., Mabeau S., Maurice M., Landrein A. (1995). Comparison of different extractive procedures for proteins from the edible seaweeds *Ulva rigida* and *Ulva rotundata*. J. Appl. Phycol..

[9] Bähr M., Fechner A., Hasenkopf K., Mittermaier S., Jahreis G. (2014). Chemical composition of dehulled seeds of selected lupin cultivars in comparison to pea and soya bean. LWT-Food Sci. Technol..

[10] Tenorio A.T., Kyriakopoulou K.E., Suarez-Garcia E., van den Berg C., van der Goot A.J. (2018). Understanding differences in protein fractionation from conventional crops, and herbaceous and aquatic biomass-consequences for industrial use. Trends Food Sci. Technol..

[11] Dawczynski C., Schubert R., Jahreis G. (2007). Amino acids, fatty acids, and dietary fibre in edible seaweed products. Food Chem..

[12] Kumar C.S., Ganesan P., Suresh P., Bhaskar N. (2008). Seaweeds as a source of nutritionally beneficial compounds—A review. J. Food Sci. Technol..

[13] Cherry P., O’Hara C., Magee P.J., McSorley E.M., Allsopp P.J. (2019). Risks and benefits of consuming edible seaweeds. Nutr. Rev..

[14] Fleurence J., Fleurence J., Levine I. (2016). Seaweeds as food. Seaweed in Health and Disease Prevention.

[15] Harnedy P.A., FitzGerald R.J., Stengel D.B., Connan S. (2015). Extraction and enrichment of protein from red and green macroalgae. Natural Products from Marine Algae: Methods and Protocols.

[16] Holdt S.L., Kraan S. (2011). Bioactive compounds in seaweed: Functional food applications and legislation. J. Appl. Phycol..

[17] Ortiz J., Romero N., Robert P., Araya J., Lopez-Hernández J., Bozzo C., Navarrete E., Osorio A., Rios A. (2006). Dietary fiber, amino acid, fatty acid and tocopherol contents of the edible seaweeds *Ulva lactuca* and *Durvillaea antarctica*. Food Chem..

[18] Patarra R.F., Paiva L., Neto A.I., Lima E., Baptista J. (2011). Nutritional value of selected macroalgae. J. Appl. Phycol..

[19] Baweja P., Kumar S., Sahoo D., Levine I., Fleurence J., Levine I. (2016). Biology of seaweeds. Seaweed in Health and Disease Prevention.

[20] Ferdouse F., Holdt S.L., Smith R., Murúa P., Yang Z. (2018). The global status of seaweed production, trade and utilization. Globefish Research Programme.

[21] Pardilhó S., Machado S., Bessada S., Almeida M., Oliveira M., Dias J. (2021). Marine macroalgae waste from Northern Portugal: A potential source of natural pigments?. Waste Biomass Valorization.

[22] Buschmann A.H., Camus C., Infante J., Neori A., Israel Á., Hernández-González M.C., Pereda S.V., Gomez-Pinchetti J.L., Golberg A., Tadmor-Shalev N. (2017). Seaweed production: Overview of the global state of exploitation, farming and emerging research activity. Eur. J. Phycol..

[23] De Bhowmick G., Hayes M. (2022). In vitro protein digestibility of selected seaweeds. Foods.

[24] Pereira L., Pomin V.H. (2011). A review of the nutrient composition of selected edible seaweeds. Seaweed: Ecology, Nutrient Composition and Medicinal Uses.

[25] Mabeau S., Fleurence J. (1993). Seaweed in food products: Biochemical and nutritional aspects. Trends Food Sci. Technol..

[26] Nunes N., Ferraz S., Valente S., Barreto M.C., De Carvalho M.P. (2017). Biochemical composition, nutritional value, and antioxidant properties of seven seaweed species from the Madeira Archipelago. J. Appl. Phycol..

[27] Harb T.B., Pereira M.S., Cavalcanti M.I.L., Fujii M.T., Chow F. (2021). Antioxidant activity and related chemical composition of extracts from brazilian beach-cast marine algae: Opportunities of turning a waste into a resource. J. Appl. Phycol..

[28] do Nascimento Santos G., do Nascimento O.S., dos Anjos Pedreira F., Ríos G.I., Vasconcelos J.N.C., de Castro Nunes J.M. (2013). Análise quali-quantitativa das algas arribadas do Norte do Estado da Bahia, Brasil. Qualitative and quantitative analysis of arribadas algae North of Bahia State, Brazil. Acta Bot. Malacit..

[29] Lourenço S.O., Barbarino E., De-Paula J.C., Pereira L.O.d.S., Marquez U.M.L. (2002). Amino acid composition, protein content and calculation of nitrogen-to-protein conversion factors for 19 tropical seaweeds. Phycol. Res..

[30] Gressler V., Fujii M.T., Martins A.P., Colepicolo P., Mancini-Filho J., Pinto E. (2011). Biochemical composition of two red seaweed species grown on the Brazilian Coast. J. Sci. Food Agric..

[31] Gressler V., Yokoya N.S., Fujii M.T., Colepicolo P., Filho J.M., Torres R.P., Pinto E. (2010). Lipid, fatty acid, protein, amino acid and ash contents in four brazilian red algae species. Food Chem..

[32] Barbot Y.N., Al-Ghaili H., Benz R. (2016). A review on the valorization of macroalgal wastes for biomethane production. Mar. Drugs.

[33] Critchley A., Hurtado A., Pereira L., Cornish M., Largo D., Paul N. (2019). Seaweed resources of the world: A 2020 vision. Bot. Mar..

[34] López-Mosquera M.E., Fernández-Lema E., Villares R., Corral R., Alonso B., Blanco C. (2011). Composting fish waste and seaweed to produce a fertilizer for use in organic agriculture. Procedia Environl. Sci..

[35] Harb T.B., Chow F. (2022). An overview of beach-cast seaweeds: Potential and opportunities for the valorization of underused waste biomass. Algal Res..

[36] Cavalcanti L.G.M.I., González Sánchez P.M., Fuji M.T. (2022). Comparison of the diversity and biomass of beach-cast seaweeds from Ne and Se Brazil. Eur. J. Phycol..

[37] Nunes N., Valente S., Ferraz S., Barreto M.C., de Carvalho M.A.P. (2020). Biochemical study of attached macroalgae from the Madeira Archipelago and beach-cast macroalgae from the Canary Islands: Multivariate analysis to determine bioresource potential. Bot. Mar..

[38] Horwitz W.A., AOAC (2003). Ash of flour. Official Methods of Analysis of the Association of Official Analytcal Chemists (AOCA).

[39] AOAC (2003). Protein (crude) in animal feed. Official Methods of Analysis of the Association of Official Analytical Chemists (AOAC).

[40] Bjarnadóttir M., Aðalbjörnsson B.V., Nilsson A., Slizyte R., Roleda M.Y., Hreggviðsson G.Ó., Friðjónsson Ó.H., Jónsdóttir R. (2018). *Palmaria palmata* as an alternative protein source: Enzymatic protein extraction, amino acid composition, and nitrogen-to-protein conversion factor. J. Appl. Phycol..

[41] Hayes M. (2020). Measuring protein content in food: An overview of methods. Foods.

[42] Bleakley S., Hayes M. (2017). Algal proteins: Extraction, application, and challenges concerning production. Foods.

[43] Mæhre H.K., Dalheim L., Edvinsen G.K., Elvevoll E.O., Jensen I.-J. (2018). Protein determination—Method matters. Foods.

[44] Barbarino E., Lourenço S.O. (2005). An evaluation of methods for extraction and quantification of protein from marine macro- and microalgae. J. Appl. Phycol..

[45] Biancarosa I., Espe M., Bruckner C., Heesch S., Liland N., Waagbø R., Torstensen B., Lock E. (2017). Amino acid composition, protein content, and nitrogen-to-protein conversion factors of 21 seaweed species from Norwegian Waters. J. Appl. Phycol..

[46] Masuko T., Minami A., Iwasaki N., Majima T., Nishimura S.-I., Lee Y.C. (2005). Carbohydrate analysis by a phenol–sulfuric acid method in microplate format. Anal. Biochem..

[47] Santa-Catarina C., Silveira V., Balbuena T.S., Viana A.M., Estelita M.E.M., Handro W., Floh E.I. (2006). IAA, ABA, polyamines and free amino acids associated with zygotic embryo development of *Ocotea catharinensis*. Plant Growth Regul..

[48] Egydio A.P.M., Santa Catarina C., Floh E.I.S., dos Santos D.Y.A.C. (2013). Free amino acid composition of *annona* (annonaceae) fruit species of economic interest. Ind. Crops Prod..

[49] Morr C.V., German B., Kinsella J.E., Regenstein J.M., Buren J.P.v., Kilara A., Lewis B.A., Mangino M.E. (1985). A collaborative study to develop a standardized food protein solubility procedure. J. Food Sci..

[50] Tamayo Tenorio A., Boom R.M., van der Goot A.J. (2017). Understanding leaf membrane protein extraction to develop a food-grade process. Food Chem..

[51] Dumay J., Morançais M., Fleurence J., Levine I. (2016). Proteins and pigments. Seaweed in Health and Disease Prevention.

[52] Vásquez V., Martínez R., Bernal C. (2019). Enzyme-assisted extraction of proteins from the seaweeds *Macrocystis pyrifera* and *Chondracanthus chamissoi:* Characterization of the extracts and their bioactive potential. J. Appl. Phycol..

[53] Harrysson H., Konasani V.R., Toth G.B., Pavia H., Albers E., Undeland I. (2019). Strategies for improving the protein yield in ph-shift processing of *Ulva lactuca* linnaeus: Effects of ulvan lyases, ph-exposure time, and temperature. ACS Sustain. Chem. Eng..

[54] Rioux L.-E., Turgeon S.L., Tiwari B.K., Troy D.J. (2015). Seaweed carbohydrates. Seaweed Sustainability.

[55] Stiger-Pouvreau V., Bourgougnon N., Deslandes E. (2016). Chapter 8 carbohydrates from seaweeds. Seaweed in Health and Disease Prevention.

[56] Hurtado A.Q., Magdugo R., Critchley A.T. (2020). Selected red seaweeds from the Philippines with emerging high-value applications. Adv. Bot. Res..

[57] Wells M.L., Potin P., Craigie J.S., Raven J.A., Merchant S.S., Helliwell K.E., Smith A.G., Camire M.E., Brawley S.H. (2017). Algae as nutritional and functional food sources: Revisiting our understanding. J. Appl. Phycol..

[58] Lahaye M. (1991). Marine algae as sources of fibres: Determination of soluble and insoluble dietary fibre contents in some ‘sea vegetables’. J. Sci. Food Agric..

[59] Lahaye M., Kaeffer B. (1997). Seaweed dietary fibres: Structure, physico-chemical and biological properties relevant to intestinal physiology. Sci. Aliments.

[60] Suzuki T., Ohsugi Y., Yoshie Y., Shirai T., Hirano T. (1996). Dietary fiber content, water-holding capacity and binding capacity of seaweeds. Fish. Sci..

[61] Mišurcová L., Kim S.-K. (2012). Chemical composition of seaweeds. Handbook of Marine Macroalgae: Biotechnology and Applied Phycology.

[62] Gelli V., Barbieri E. (2015). Cultivo e aproveitamento da macroalga *Kappaphycus alvarezii* para pequenos maricultores. Aquicultura no Brasil: Novas perspectivas. Pedro João.

[63] Rupérez P. (2002). Mineral content of edible marine seaweeds. Food Chem..

[64] Kraan S. (2013). Pigments and minor compounds in algae. Functional Ingredients from Algae for Foods and Nutraceuticals.

[65] Kato H., Rhue M.R., Nishimura T. (1989). Role of free amino acids and peptides in food taste. Flavor Chem..

[66] Fleurence J., Yada R.Y. (2004). Seaweed proteins. Proteins in Food Processing.

[67] Harnedy P.A., FitzGerald R.J. (2011). Bioactive proteins, peptides, and amino acids from macroalgae. J. Phycol..

[68] Conde E., Balboa E.M., Parada M., Falqué E., Domínguez H. (2013). Algal proteins, peptides and amino acids. Functional Ingredients from Algae for Foods and Nutraceuticals.

[69] MacArtain P., Gill C.I.R., Brooks M., Campbell R., Rowland I.R. (2007). Nutritional value of edible seaweeds. Nutr. Rev..

[70] Kumari P., Kumar M., Gupta V., Reddy C.R.K., Jha B. (2010). Tropical marine macroalgae as potential sources of nutritionally important PUFAs. Food Chem..

[71] Harrysson H., Hayes M., Eimer F., Carlsson N.G., Toth G.B., Undeland I. (2018). Production of protein extracts from swedish red, green, and brown seaweeds, *Porphyra umbilicalis* (Kutzing), *Ulva lactuca* (Linnaeus), and *Saccharina latissima* (Linnaeus) j. V. Lamouroux using three different methods. J. Appl. Phycol..

[72] Sánchez-Machado D.I., López-Cervantes J., López-Hernández J., Paseiro-Losada P. (2004). Fatty acids, total lipid, protein and ash contents of processed edible seaweeds. Food Chem..

[73] Fleurence, Massiani L., Guyader O., Mabeau S. (1995). Use of enzymatic cell wall degradation for improvement of protein extraction from *Chondrus crispus*, *Gracilaria verrucosa* and *Palmaria palmata*. J. Appl. Phycol..

[74] Vilg J.V., Undeland I. (2017). Ph-driven solubilization and isoelectric precipitation of proteins from the brown seaweed *Saccharina latissima*—effects of osmotic shock, water volume and temperature. J. Appl. Phycol..

[75] Muranyi I.S., Otto C., Pickardt C., Osen R., Koehler P., Schweiggert-Weisz U. (2016). Influence of the isolation method on the technofunctional properties of protein isolates from *Lupinus angustifolius* L.. J. Food Sci..

[76] Cornish M.L., Mouritsen O.G., Critchley A.T. (2019). A mini-review on the microbial continuum: Consideration of a link between judicious consumption of a varied diet of macroalgae and human health and nutrition. J. Ocean Limnol..

