# Supplementary material for: Nutritional Composition of Beach-Cast Marine Algae from the Brazilian Coast: Added Value for Algal Biomass Considered as Waste
Source: Foods. 2022 Apr 21;11(9):1201. doi: 10.3390/foods11091201 (PMC9099717; doi:10.3390/foods11091201)
Supplement: Supplementary file 1 [file foods-11-01201-s001.zip › foods-1505295-supplementary.pdf]

SUPPLEMENTARY MATERIAL

Table S1. Summary of the beach-cast algae collected from southeast and northeast beaches, Brazil. CE: Ceará State, northeast coast. ES: Espírito Santo State, southeast coast. PE: Pernambuco State, northeast coast.

Species	Beach (State)	Localization	Nº Voucher (herbarium)	Data of collection
**Rhodophyta (red algae)**				
Agardhiella ramosissima (Harvey) Kylin	Itaoca Beach (ES)	20°54'18.0"S; 40°46'42.3"W	SP470206	30/04/2018
Alsidium seaforthii (Turner) J. Agardh	Piúma Beach (ES)	20°50'31.5"S; 40°43'46.0"W	SPF58253	11/06/2018
Alsidium triquetrum (S.G. Gmelin) Trevisan	Emboaca Beach (CE)	3°12'23.5"S; 39°18'37.1"W	SPF58318	30/03/2018
Botryocladia occidentalis (Børgesen) Kylin	Emboaca Beach (CE)	3°12'23.5"S; 39°18'37.1"W	SPF58317	30/03/2018
Gracilaria domingensis (Kützing) Sonder ex Dickie	Emboaca Beach (CE)	3°12'23.5"S; 39°18'37.1"W	SPF58316	30/03/2018
Halymenia brasiliana S.M.P.B. Guimarães & M.T. Fujii	Itaoca Beach (ES)	20°54'18"S; 40°46'42.3"W	SP470204	30/04/2018
Osmundaria obtusiloba (C. Agardh) R.E. Norris	Piúma Beach (ES)	20°50'31.5"S; 40°43'46.0"W	SPF58344	11/06/2018
Spyridia clavata Kützing	Pontal Beach (ES)	20°58'22.5"S; 40°48'38.6"W	SPF58251	09/06/2017
**Phaeophyceae (brown algae)**				
Dictyopteris jolyana E.C. Oliveira & R.P. Furtado	Pontal Beach (ES)	20°58'22.5"S; 40°48'38.6"W	SPF58249	30/04/2018
Spatoglossum schroederi (C. Agardh) Kützing	Candeias Beach (PE)	8°12'46"S; 34°55'6"W	SP470200	25/02/2018
Zonaria tournefortii (J.V. Lamouroux) Montagne	Pontal Beach (ES)	20°58'22.5"S; 40°48'38.6"W	SPF58252	09/06/2017
**Chlorophyta (green algae)**				
Codium isthmocladum Vickers	Itaoca Beach (ES)	20°54'18.0"S; 40°46'42.3"W	SP470207	30/04/2018

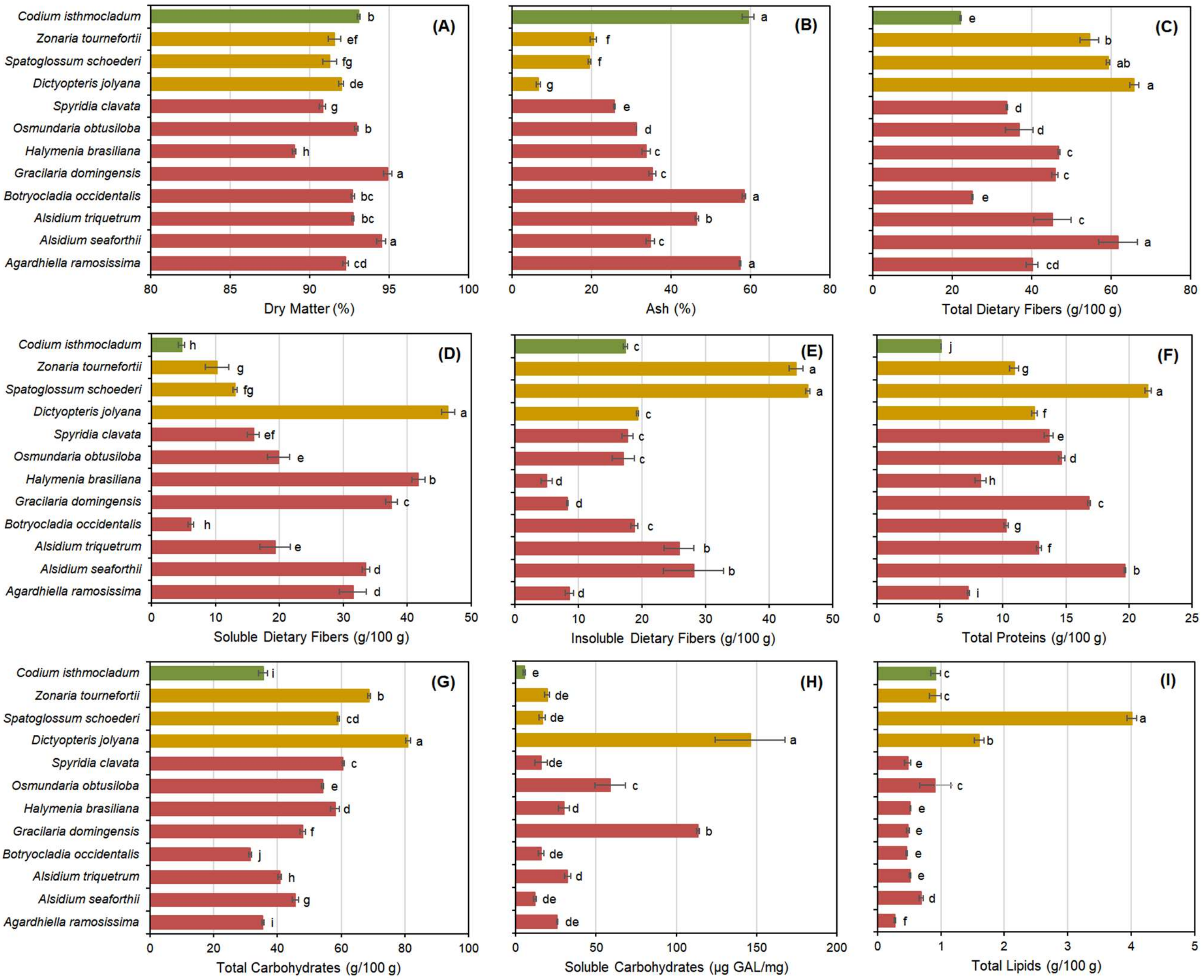

FIGURE S1. Proximate composition of beach-cast macroalgae. Values represent the average of three replicates (mean ± SD), and letters indicate the statistical significance ($p < 0.05$).

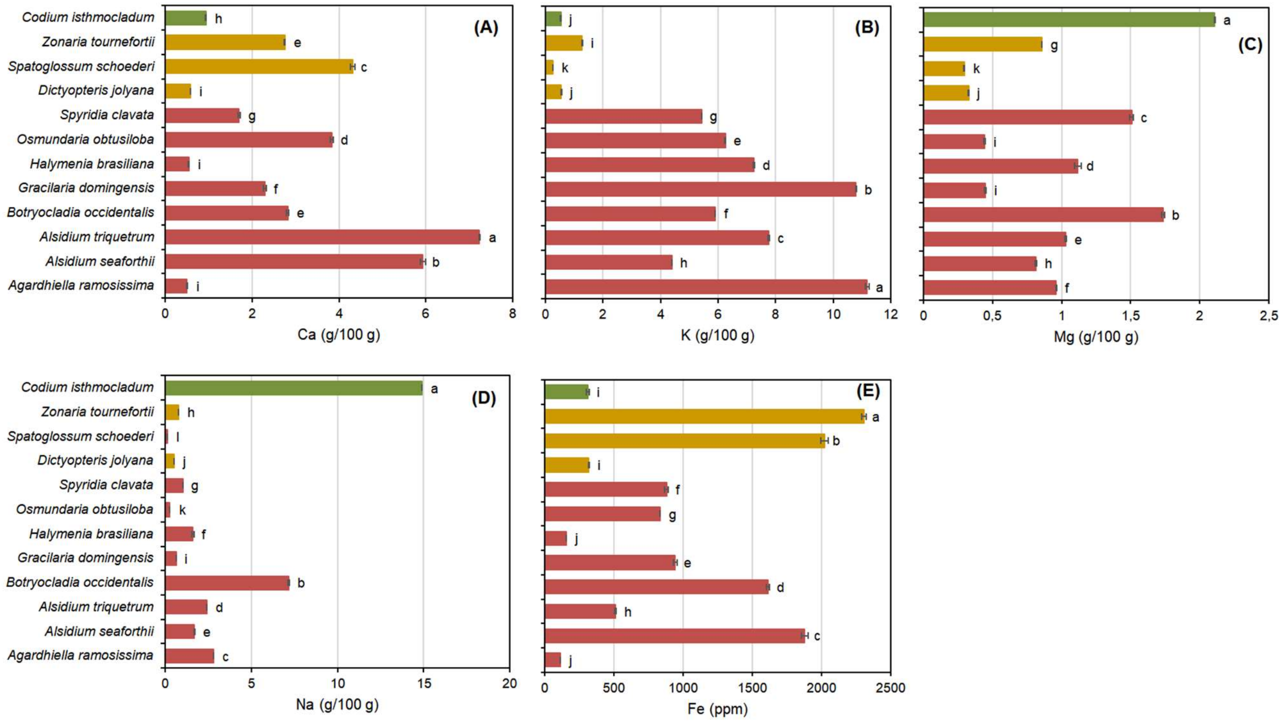

FIGURE S2. Macro (N, P, Ca, K, Mg, and Na) and micro (Fe) elements as well as trace metals of beach-cast macroalgae. Values represent the average of three technical replicates (mean ± SD), and letters indicate the statistical significance ($p < 0.05$).

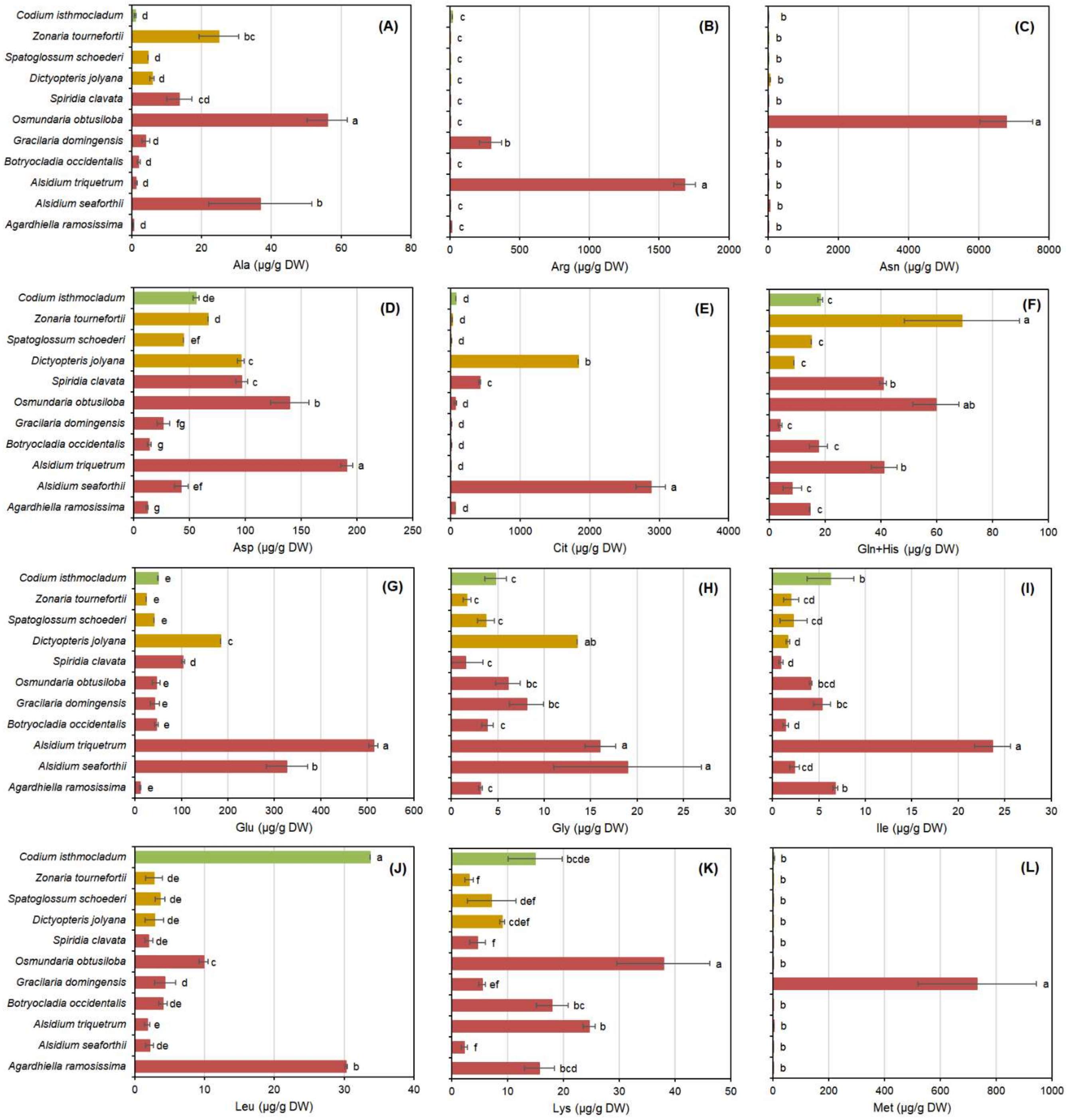

FIGURE S3. Free Amino acids composition of beach-cast macroalgae. Values represent the average of three technical replicates (mean ± SD), and letters indicate the statistical significance ($p < 0.05$).

(continued...)

FIGURE S3 (continued...)

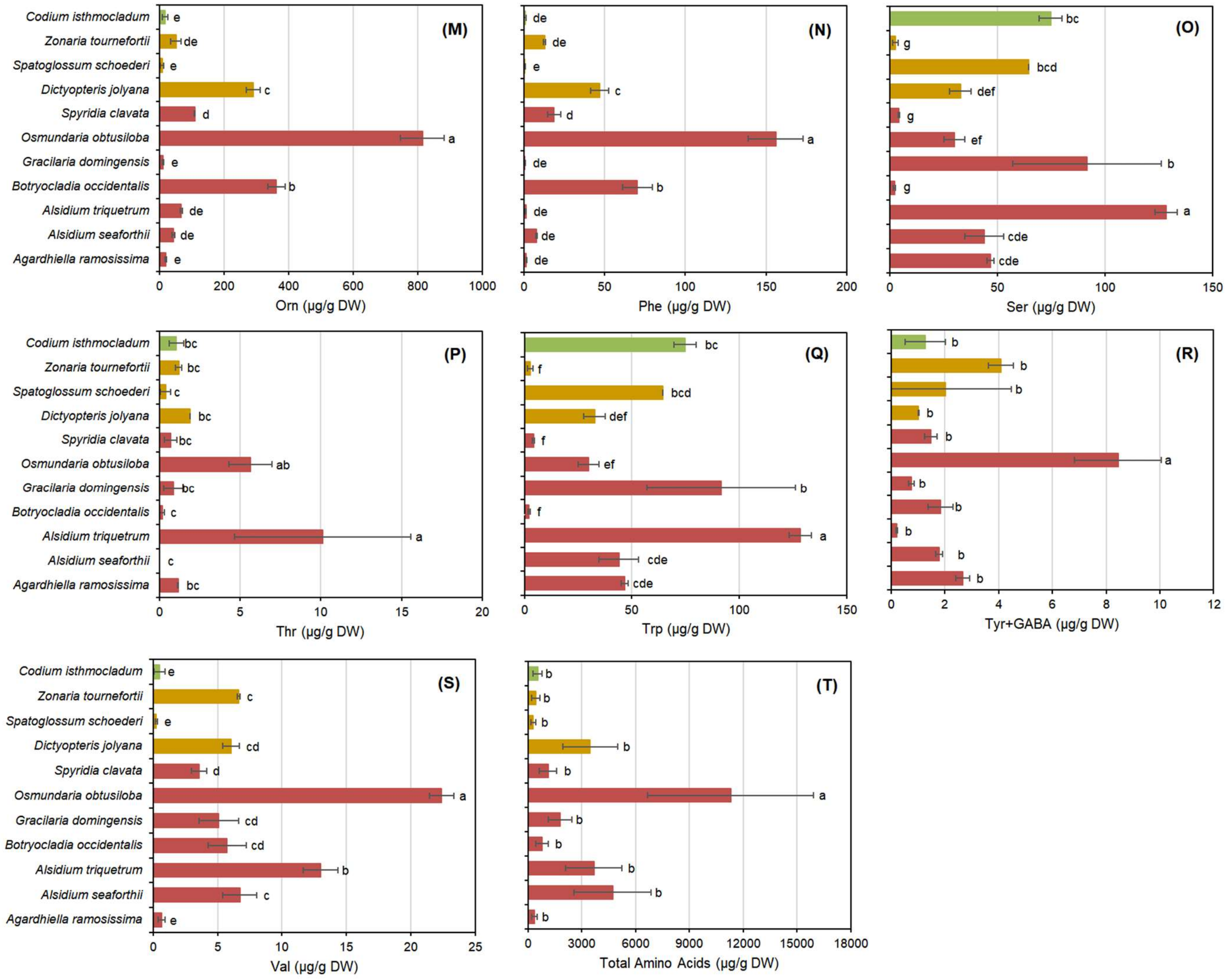

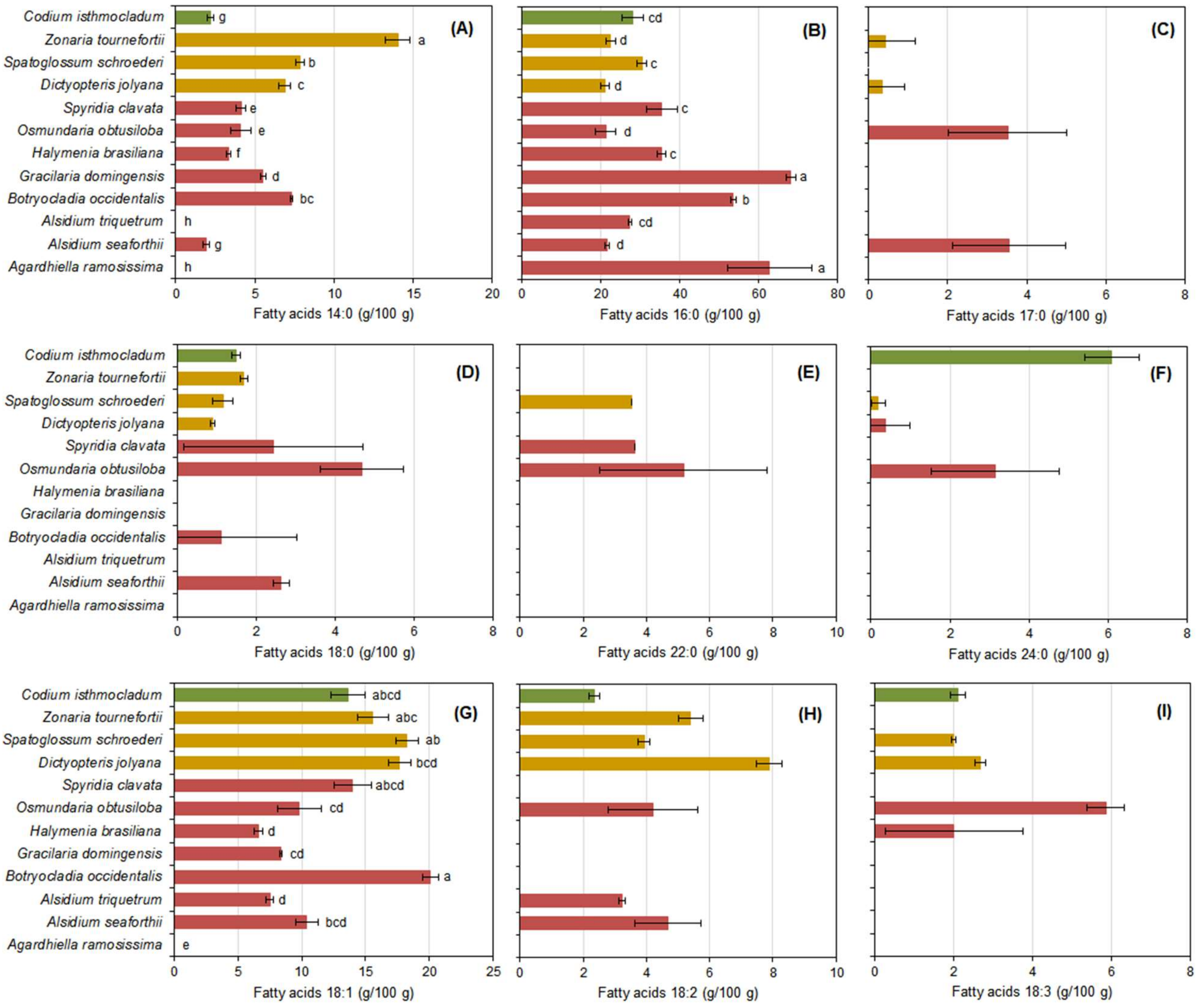

FIGURE S4. Fatty acids composition of beach-cast macroalgae. Values represent the average of three replicates (mean ± SD), and letters indicate the statistical significance ($p < 0.05$). Statistical analysis was conducted only for amounts over 10.

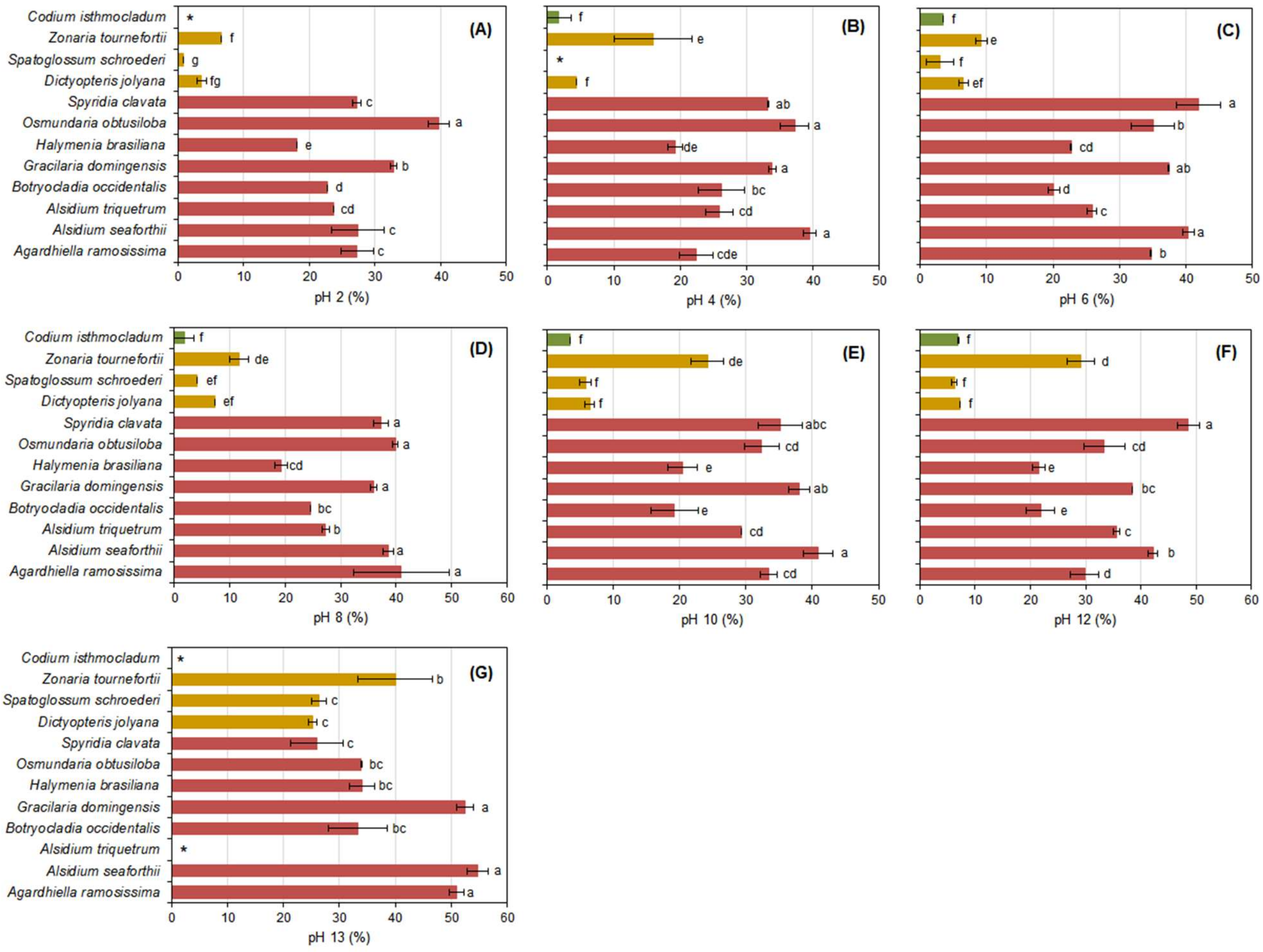

FIGURE S5. Solubility of total proteins from beach-cast macroalgae at different pH levels. Values represent the average of three replicates (mean ± SD) and letters indicate the statistical significance ($p < 0.05$).

Table S2. Calculation of nitrogen-to-protein conversion factors for twelve beach-cast macroalgae on the amino acid residues to total nitrogen ration. (µg/100 g = % on dry mass basis). Values represent the average of three replicates (mean ± SD).

Species	Total Proteins (Nx6.25)	Total Proteins (Nxspecies-specific factors)[a]
**Rhodophyta (red algae)**		
Agardhiella ramosissima	7.3 ± 0.1	4.6 ± 0.1
Alsidium seaforthii	19.7 ± 0.1	12.6 ± 0.1
Alsidium triquetrum	12.8 ± 0.2	8.2 ± 0.2
Botryocladia occidentalis	10.3 ± 0.2	6.5 ± 0.2
Gracilaria domingensis	16.8 ± 0.1	10.8 ± 0.1
Halymenia brasiliana	8.2 ± 0.4	5.3 ± 0.4
Osmundaria obtusiloba	14.6 ± 0.2	9.3 ± 0.3
Spyridia clavata	13.6 ± 0.2	8.7 ± 0.3
**Phaeophyceae (brown algae)**		
Dictyopteris jolyana	12.5 ± 0.2	10.3 ± 0.2
Spatoglossum schroederi	21.5 ± 0.2	17.7 ± 0.2
Zonaria tournefortii	10.9 ± 0.4	8.9 ± 0.3
**Chlorophyta (green algae)**		
Codium isthmocladum	5.1 ± 0.1	3.5 ± 0.0

[a] by Biancarosa et al. (2017) nitrogen-to-protein conversion factor of 5.13 ± 0.1 for brown, 3.99 ± 0.39 for red, and 4.24 ± 0.46 for green seaweeds.
